# A Novel Scoring System for Response of Preoperative Chemoradiotherapy in Locally Advanced Rectal Cancer Using Early-Treatment Blood Features Derived From Machine Learning

**DOI:** 10.3389/fonc.2021.790894

**Published:** 2021-11-29

**Authors:** Jaesik Kim, Kyung-Ah Sohn, Jung-Hak Kwak, Min Jung Kim, Seung-Bum Ryoo, Seung-Yong Jeong, Kyu Joo Park, Hyun-Cheol Kang, Eui Kyu Chie, Sang-Hyuk Jung, Dokyoon Kim, Ji Won Park

**Affiliations:** ^1^ Department of Computer Engineering, Ajou University, Suwon, South Korea; ^2^ Department of Biostatistics, Epidemiology & Informatics, Perelman School of Medicine, University of Pennsylvania, Philadelphia, PA, United States; ^3^ Institute for Biomedical Informatics, University of Pennsylvania, Philadelphia, PA, United States; ^4^ Department of Artificial Intelligence, Ajou University, Suwon, South Korea; ^5^ Department of Surgery, Seoul National University College of Medicine, Seoul National University Hospital, Seoul, South Korea; ^6^ Cancer Research Institute, Seoul National University, Seoul, South Korea; ^7^ Department of Radiation Oncology, Seoul National University College of Medicine, Seoul, South Korea; ^8^ Institute of Radiation Medicine, Medical Research Center, Seoul National University, Seoul, South Korea; ^9^ Department of Digital Health, Samsung Advanced Institute for Health Sciences & Technology (SAIHST), Sungkyunkwan University, Samsung Medical Center, Seoul, South Korea

**Keywords:** machine learning, preoperative chemoradiotherapy, rectal cancer, pathologic response, early-treatment blood features, prediction

## Abstract

**Background:**

Preoperative chemoradiotherapy (CRT) is a standard treatment for locally advanced rectal cancer (LARC). However, individual responses to preoperative CRT vary from patient to patient. The aim of this study is to develop a scoring system for the response of preoperative CRT in LARC using blood features derived from machine learning.

**Methods:**

Patients who underwent total mesorectal excision after preoperative CRT were included in this study. The performance of machine learning models using blood features before CRT (pre-CRT) and from 1 to 2 weeks after CRT (early-CRT) was evaluated. Based on the best model, important features were selected. The scoring system was developed from the selected model and features. The performance of the new scoring system was compared with those of systemic inflammatory indicators: neutrophil-to-lymphocyte ratio, platelet-to-lymphocyte ratio, lymphocyte-to-monocyte ratio, and the prognostic nutritional index.

**Results:**

The models using early-CRT blood features had better performances than those using pre-CRT blood features. Based on the ridge regression model, which showed the best performance among the machine learning models (AUROC 0.6322 and AUPRC 0.5965), a novel scoring system for the response of preoperative CRT, named Response Prediction Score (RPS), was developed. The RPS system showed higher predictive power (AUROC 0.6747) than single blood features and systemic inflammatory indicators and stratified the tumor regression grade and overall downstaging clearly.

**Conclusion:**

We discovered that we can more accurately predict CRT response by using early-treatment blood data. With larger data, we can develop a more accurate and reliable indicator that can be used in real daily practices. In the future, we urge the collection of early-treatment blood data and pre-treatment blood data.

## 1 Introduction

Rectal cancer represents approximately one-third of all colorectal cancer with the third highest incidence (1). A considerable proportion (about 30–40%) of rectal cancer is locally advanced rectal cancer (LARC) (2, 3). Local recurrence rates of rectal cancer are relatively higher than those of colon cancer. To reduce local recurrence in rectal cancer, radiotherapy has been performed in locally advanced rectal cancer (LARC). In particular, patients treated with preoperative radiotherapy have fewer local recurrences than those treated with postoperative radiotherapy (4). Currently, preoperative radiotherapy or chemoradiotherapy (CRT) is accepted as a standard of care for rectal cancer (5). For identification of LARC, the pretreatment staging is evaluated using computed tomography of the abdomen and pelvis, rectal magnetic resonance imaging, and/or transrectal ultrasound. As a neoadjuvant treatment of LARC, long-course CRT is given as concurrent fluoropyrimidine-based chemotherapy combined with radiotherapy over 5.5 to 6 weeks. Total mesorectal excision is performed commonly 6 to 8 weeks after the completion of long-course CRT. The response to CRT is assessed by pathologic examination after surgery. However, individual response to CRT is variable across patients. Although about 10–20% of patients have a pathologic complete response, up to 30% of patients have no response to CRT (6, 7).

According to the response to CRT, tailored treatments can be applied. In patients with complete response, “wait and watch” or local excision can be one of the treatment options. In non-responders, an ineffective treatment could be avoided. For these patients, other therapeutic approaches, such as surgery, intensified radiotherapy, or systemic chemotherapy, can be applied after stopping CRT. In this fashion, prediction of response can be used for the stratification of patients with LARC. No robust predictive markers or models for response to CRT have been identified at present. Routinely tested pre-treatment blood indicators have recently attracted attention as new predictive indicators. Systemic inflammatory and nutritional indicators, namely, neutrophil-to-lymphocyte ratio (NLR), platelet-to-lymphocyte ratio (PLR), lymphocyte-to-monocyte ratio (LMR), and the prognostic nutritional index (PNI), have the potential to predict the response to CRT (8, 9). These indicators were made using pre-treatment blood features. Futhermore, blood features at the early phase during treatment may give more information on the response of CRT than those before treatment (10). Early-treatment predictive indicators can help clinicians to decide whether to continue CRT or not. However, few studies have investigated the predictive value of early-treatment blood features.

**Figure 1:**
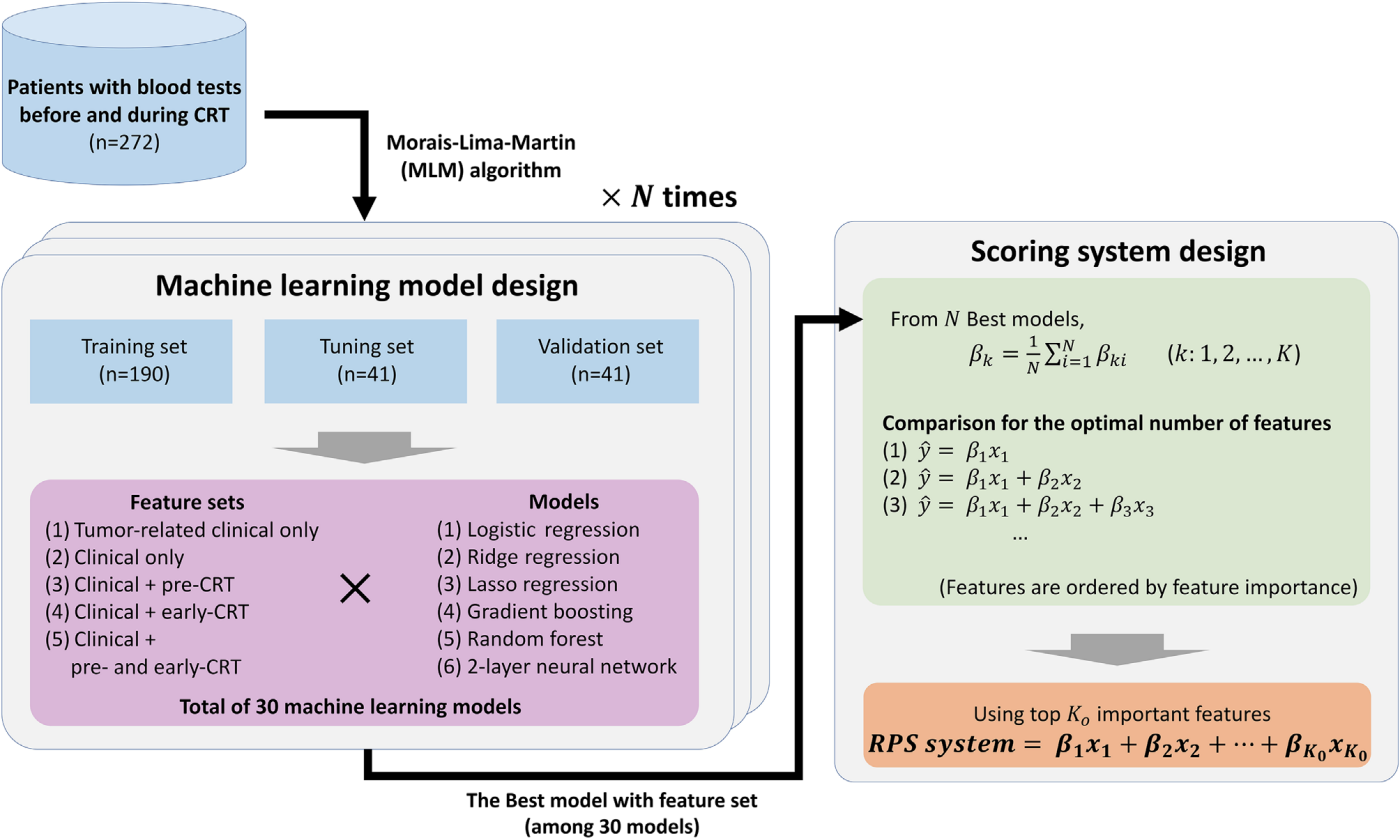
Overview of the entire study. The best model is selected by evaluating possible machine learning models with *N* repeats, and a novel scoring system with significant predictive power and simplicity is designed using the *N* best models. The order of important features is determined based on generalized feature importance (*β_k_*), and significance of score candidates are compared to find the optimal number of important features (*K_o_*).

In this study, we verified the hypothesis that blood features at the early phase during treatment include more significant information, by evaluating the predictive value of pre-treatment and early-treatment blood features for the response of preoperative CRT in LARC. We compared a total of 30 machine learning models by the combination of six types of machine learning models and five combinations of feature sets. Moreover, we developed a novel scoring system, named Response Prediction Score (RPS) using the feature importance of the best model among the 30 models. The study design is described in **Figure 1**. The RPS system outperformed both pre-treatment and early-treatment indicators using single blood features and systemic inflammatory and nutritional indicators. In daily clinical practice, the RPS system can be used to assist the clinician’s decision to “continue or stop” at the early phase during CRT. To extend our findings, we urge the collection of early-treatment blood data and pre-treatment blood data to develop a more accurate and reliable indicator that can be used in real daily practices in the future.

## 2 Materials and Methods

### 2.1 Materials

#### 2.1.1 Dataset

Patients who underwent total mesorectal excision with preoperative CRT for LARC in the Seoul National University Hospital from July 2002 to June 2019 were eligible for this study. All patients had histologically confirmed adenocarcinoma of the rectum, which was located below 15 cm from the anal verge. Approximately 272 patients, who had the results of blood tests before or during CRT, were included in this study. The study was conducted according to the guidelines of the Declaration of Helsinki, and approved by the institutional review board of the Seoul National University Hospital (No. 1606-037-768). Patient consent was waived because of the retrospective nature of the study.

#### 2.1.2 Treatment

The median dose of total radiotherapy was 50.4 Gy, delivered as 1.8 Gy per fraction, with 45 Gy in 25 fractions to the large field of the pelvis followed by 5.4 Gy in 3–6 fractions to the reduced field of the primary lesion, threatened tumor margin, and enlarged lateral lymph nodes. For concurrent chemotherapy with radiotherapy, 5-fluorouracil or capecitabine was administered. Following CRT, patients underwent total mesorectal excision 5 to 12 weeks after CRT.

#### 2.1.3 Evaluation

Before CRT, history taking, physical examination and distal rectal examination were done. Colonoscopic biopsy, blood tests, and computed tomography of the abdomino-pelvis and chest were performed. Rectal magnetic resonance imaging was performed in most patients. To assess pathologic responses of CRT, postoperative specimens were examined by pathologists. All pathologic specimens were examined by experienced gastrointestinal pathologists. The pathologic responses were categorized into four tiers using the American Joint Committee on Cancer (AJCC) tumor regression grade (TRG) system (11). The TRG system was defined as follows: TRG 0 was defined as no viable cancer cells (complete response); TRG 1 was defined as single or small groups of tumor cells (moderate response); TRG 2 was defined as residual cancer outgrown by fibrosis (minimal response); and TRG 3 was defined as minimal or no tumor cells killed (poor response). A good responder was defined as a patient with TRG 0 and 1, and a poor responder was defined as a patient with TRG 2 and 3.

#### 2.1.4 Blood Measurements

The following blood features were obtained for each patient before CRT (pre-CRT): complete blood counts with differentiation (red blood cell count [RBC], hemoglobin [HB], hematocrit [Hct], mean red cell volume [MCV], mean red cell hemoglobin [MCH], mean red blood cell hemoglobin content [MCHC], red blood cell distribution width [RDW], platelet [PLT], plateletcrit [PCT], mean platelet volume [MPV], platelet distribution width [PDW], white blood cell [WBC], neutrophil, lymphocyte, monocyte, eosinophil and basophil count), blood chemistry tests (calcium, phosphorus, glucose, uric acid, cholesterol, total protein, albumin, total bilirubin, aspartate transaminase [AST], alanine aminotransferase [ALT], alkaline phosphatase, creatinine), and carcinoembryonic antigen (CEA) level. Complete blood counts with differentiation were measured from 1 to 2 weeks after CRT (early-CRT). Laboratory index values that had previously been reported in association with systemic inflammation and nutrition were calculated: NLR (*neutrophil count/lymphocyte count*), PLR (*platelet count/lymphocyte count*), LMR (*lymphocyte count/monocyte count*) and PNI (10 × *serum albumin* (*g/dL*) + 0.005 × *total lymphocyte count* (/*mm* ^3^) (12–15).

### 2.2 Machine Learning Model

#### 2.2.1 Data Splitting Algorithm

In machine learning approaches, cross-validation by data splitting is necessary in order to construct a stable and confident model and avoid overfitting. Random selection-based data splitting has the advantage of simplicity. However, due to the small sample size of the biomedical data, heterogeneity between the training and testing datasets can be induced by improper splitting, which can become a fatal weakness in modeling. To avoid this splitting bias, the Kennard Stone (KS) algorithm (16, 17) limits most sources of variations within the dataset into training models, ensuring that the training model better represents the entire data. After data standardization, the KS algorithm selects the sample with the maximum distance from all other samples, then repeats the process of selecting the next sample as far away as possible from the selected sample until the selected number of samples is reached. This way leads the selected samples to cover the entire sample space uniformly without selection bias, and the selected samples will be used for the training dataset and the others will be used for the testing dataset. However, the KS algorithm has a limitation of having a small degree of randomness, and a developed method with more randomness was introduced, which is called the Morais–Lima–Martin (MLM) algorithm (18). The MLM algorithm applies a random-mutation factor to the KS results, where some samples from the training set are transferred to the testing set, and some samples from the testing set are transferred to the training set. The introduced mutation rate was set at 10%.

#### 2.2.2 Model Design

After splitting the data by the MLM algorithm into 70% training set, 15% tuning set, and 15% validation set, we proceeded with modeling using five different feature sets, namely, tumor-related clinical only (distant from anal verge, CEA, and tumor grade), clinical only, clinical plus pre-CRT, clinical plus early-CRT, and clinical plus pre-CRT and early-CRT, with six machine learning models: logistic regression, ridge regression, lasso regression (linear models), gradient boosting, random forest (tree-based ensemble models), and a two-layer neural network (neural network model), for the response of preoperative CRT prediction. In other words, a total of 30 types of predictive models were learned and compared with their performances on the validation set. During learning, each model determined optimal hyperparameter values that led to the best performance on the tuning set within the hyperparameter search space (**Supplementary Table 3**). For a generalization, we repeated *N* times data splitting, model learning, and evaluation.

#### 2.2.3 Feature Selection

Through our preliminary experiment, we found that the prediction performance of using entire available features was poor. Therefore, all the models except the lasso model had a feature selection step first. Significant features were selected by a certain P-value threshold from a Mann–Whitney U test and a Chi-squared test on the training set, and the model was trained with these features. In our experiment, we selected P-value <0.1. The two-layer neural network does not apply feature selection step because it involves the ability of feature extraction instead of selection.

### 2.3 Feature Importance and New Scoring System

Different feature importance from *N* models can be obtained from repetition. We were able to find generalized feature importance (*β_k_*) through averaging feature importance from the *N* best models (Equation 1). By statistical testing, we first found the top *K_o_* most important features, and then defined a simple and powerful scoring system, called the Response Prediction Score (RPS), which is used for predicting preoperative CRT response. With this, we checked if the same feature of 1,000 models exhibits a consistent sign of the coefficient. To evaluate RPS, we compared RPS with single blood features and inflammatory-nutritional indicators; NLR, PLR, LMR, and PNI.


(1) $$\beta_{k}=\frac{1}{N}\sum_{i=1}^{N}\beta_{ki}\;(k:\;1,2\ldots ,K)$$

where *β_k_* denotes generalized feature importance, and *K* denotes the number of all features.

To evaluate the versatility of RPS, prediction of the probability for tumor downstaging was investigated. Overall downstaging, T-downstaging, and N-downstaging were examined for tumor downstaging. Overall downstaging was defined as ypT0-2N0M0 from pathologic examination after surgery. T-downstaging was defined as the lowering of T classification from clinical T classification to pathologic T classification. N-downstaging was defined as the lowering of N classification from clinical N classification to pathologic N classification.

### 2.4 Performance Metric

A receiver operating characteristic (ROC) curve is widely used for evaluating prediction models. It plots the True Positive Rate (TPR) against the False Positive Rate (FPR). The **AUROC** stands for the area under the ROC curve. A Precision-Recall curve (PRC) is another evaluation method. It plots precision against recall. The **AUPRC** denotes the area under the PR curve. TPF, FPR, Precision, and Recall are defined as follows:


(2) $$\text{TPR}(=\text{Sensitivity}=\text{Recall})=\frac{\text{TP}}{\text{TP}+\text{FN}}$$

(3) $$\text{FPR}(=1-\text{Specificity})=\frac{\text{FP}}{\text{FP}+\text{TN}}$$

(4) $$\text{Precision}=\frac{\text{TP}}{\text{TP}+\text{FP}}$$

where TP, FP, TN, and FN are the number of true positives, false positives, true negatives, and false negatives, respectively. These evaluation metrics allow us to compare the prediction models more formally and precisely.

### 2.5 Statistical Test

A Chi-squared test was used for categorical variables and a Mann–Whitney U test was used for continuous variables. A two-sided P-value of <0.05 was considered statistically significant. Analyses were performed using Python (version 3.7.3, Python Software Foundation, Beaverton, USA).

## 3 Results

### 3.1 Model Performance

During machine learning model training, label-balance of the dataset was almost equally balanced: training set 82.5 (43.4%)/107.5 (56.6%) tuning set 19.1 (46.6%)/21.9 (53.4%), and validation set 19.4 (47.3%)/21.6 (52.7%) (the average number of responders/non-responders of 1,000 repeats). The mean and standard deviation of the results from repeating a total of 30 models 1,000 times were obtained (**Table 1**). For tumor-related clinical only and clinical only feature sets, all features were removed in the training step while feature selection was used in most iterations during the 1,000 repeats. Therefore, we did not apply the feature selection method in this experiment. First, compared to the models using five different types of feature sets, the result for tumor-related clinical only and clinical only feature sets showed poor AUROC and AUPRC performance close to 0.5. The models with early-CRT showed better performance than the models with pre-CRT or pre-CRT plus early-CRT in all types of models. The models with pre-CRT showed a performance close to random prediction, given that their AUROCs and AUPRCs showed values close to 0.5, which shows features in the pre-CRT set have little important information in solving tasks. Therefore, the performance of the models with pre-CRT plus early-CRT was also lower than that of early-CRT only.

**Table 1:** Performance comparison of a total of 18 models for 1,000 repeats.

	Training set		Tuning set		Validation set	
	AUROC (mean ± std)	AUPRC (mean ± std)	AUROC (mean ± std)	AUPRC (mean ± std)	AUROC (mean ± std)	AUPRC (mean ± std)
**Tumor-related clinical only**						
Logistic regression	0.6212 ± 0.0206	0.5352 ± 0.0279	0.5448 ± 0.0835	0.5301 ± 0.1062	0.5353 ± 0.0785	0.5325 ± 0.1039
Ridge regression	0.6210 ± 0.0205	0.5353 ± 0.0280	0.5458 ± 0.0836	0.5313 ± 0.1063	0.5339 ± 0.0784	0.5312 ± 0.1039
Lasso regression	0.5857 ± 0.0521	0.5762 ± 0.0848	0.5687 ± 0.0653	0.6035 ± 0.1137	0.5133 ± 0.0634	0.5675 ± 0.1311
Gradient boosting	0.9524 ± 0.0731	0.9479 ± 0.0776	0.5743 ± 0.0784	0.5369 ± 0.1035	0.5034 ± 0.0806	0.4917 ± 0.0956
Random forest	0.8594 ± 0.1227	0.8461 ± 0.1341	0.5791 ± 0.0775	0.5352 ± 0.1023	0.5123 ± 0.0774	0.4942 ± 0.0940
Two-layer neural network	0.6031 ± 0.0510	0.5366 ± 0.0441	0.6166 ± 0.0724	0.5922 ± 0.1077	0.5175 ± 0.0812	0.5144 ± 0.1006
**Clinical only**						
Logistic regression	0.6529 ± 0.0210	0.5593 ± 0.0282	0.5117 ± 0.0844	0.4875 ± 0.0993	0.5081 ± 0.0788	0.4961 ± 0.0974
Ridge regression	0.6487 ± 0.0205	0.5572 ± 0.0283	0.5303 ± 0.0830	0.5056 ± 0.1021	0.5108 ± 0.0787	0.5044 ± 0.0999
Lasso regression	0.5933 ± 0.0637	0.5956 ± 0.0824	0.5586 ± 0.0605	0.5995 ± 0.1178	0.4957 ± 0.0623	0.5587 ± 0.1404
Gradient boosting	0.9938 ± 0.0191	0.9930 ± 0.0217	0.5825 ± 0.0745	0.5432 ± 0.1039	0.5027 ± 0.0821	0.4972 ± 0.1001
Random forest	0.9779 ± 0.0517	0.9737 ± 0.0621	0.5858 ± 0.0780	0.5475 ± 0.1066	0.5280 ± 0.0812	0.5193 ± 0.1011
Two-layer neural network	0.8048 ± 0.1393	0.7687 ± 0.1628	0.6094 ± 0.0675	0.5726 ± 0.1025	0.5089 ± 0.0878	0.5068 ± 0.1010
**Clinical + pre-CRT**						
Logistic regression w/FS	0.6463 ± 0.0286	0.5770 ± 0.0348	0.4881 ± 0.0815	0.4772 ± 0.0954	0.4856 ± 0.0854	0.4852 ± 0.0955
Ridge regression w/FS	0.6450 ± 0.0276	0.5753 ± 0.0335	0.4954 ± 0.0810	0.4820 ± 0.0954	0.4827 ± 0.0868	0.4832 ± 0.0955
Lasso regression	0.6983 ± 0.1125	0.6915 ± 0.0604	0.5764 ± 0.0647	0.5961 ± 0.1110	0.5022 ± 0.0715	0.5471 ± 0.1293
Gradient boosting w/FS	0.9848 ± 0.0413	0.9815 ± 0.0489	0.5757 ± 0.0825	0.5392 ± 0.1052	0.4983 ± 0.0916	0.4919 ± 0.1028
Random forest w/FS	0.9338 ± 0.0967	0.9193 ± 0.1173	0.5502 ± 0.0840	0.5194 ± 0.1035	0.4910 ± 0.0893	0.4856 ± 0.0998
Two-layer neural network	0.6937 ± 0.1320	0.6416 ± 0.1496	0.5827 ± 0.0703	0.5531 ± 0.0989	0.4917 ± 0.0870	0.4879 ± 0.0955
**Clinical + early-CRT**						
Logistic regression w/FS	0.6870 ± 0.0243	0.6032 ± 0.0281	0.5894 ± 0.0864	0.5508 ± 0.1087	0.5975 ± 0.0804	0.5681 ± 0.1052
Ridge regression w/F	0.6711 ± 0.0227	0.5845 ± 0.0281	0.6400 ± 0.0800	0.5959 ± 0.1087	0.6322 ± 0.0771	0.5965 ± 0.1067
Lasso regression	0.6923 ± 0.0568	0.6357 ± 0.0415	0.6285 ± 0.0764	0.5913 ± 0.1082	0.5844 ± 0.0815	0.5663 ± 0.1113
Gradient boosting w/FS	0.9911 ± 0.0171	0.9898 ± 0.0194	0.5968 ± 0.0738	0.5616 ± 0.1027	0.5226 ± 0.0897	0.5160 ± 0.1034
Random forest w/FS	0.8629 ± 0.0948	0.8395 ± 0.1120	0.6189 ± 0.0763	0.5830 ± 0.1075	0.5857 ± 0.0836	0.5723 ± 0.1087
Two-layer neural network	0.7053 ± 0.0879	0.6484 ± 0.1040	0.6514 ± 0.0770	0.6126 ± 0.1084	0.5836 ± 0.0901	0.5636 ± 0.1084
**Clinical + pre- and early-CRT**						
Logistic regression w/FS	0.7145 ± 0.0241	0.6283 ± 0.0315	0.5690 ± 0.0837	0.5303 ± 0.1025	0.5742 ± 0.0840	0.5451 ± 0.1036
Ridge regression w/F	0.6982 ± 0.0228	0.6023 ± 0.0302	0.6242 ± 0.0807	0.5734 ± 0.1064	0.6151 ± 0.0819	0.5757 ± 0.1060
Lasso regression	0.7118 ± 0.0857	0.6697 ± 0.0677	0.6131 ± 0.0718	0.5862 ± 0.1058	0.5610 ± 0.0856	0.5558 ± 0.1134
Gradient boosting w/FS	0.9970 ± 0.0079	0.9965 ± 0.0091	0.6265 ± 0.0759	0.5875 ± 0.1038	0.5581 ± 0.0921	0.5427 ± 0.1065
Random forest w/FS	0.9295 ± 0.0772	0.9114 ± 0.0979	0.6300 ± 0.0789	0.5825 ± 0.1064	0.5918 ± 0.0830	0.5630 ± 0.1078
Two-layer neural network	0.8001 ± 0.1160	0.7583 ± 0.1411	0.6420 ± 0.0741	0.6015 ± 0.1054	0.5622 ± 0.0882	0.5346 ± 0.1042

w/FS denotes “with feature selection”.

Next, the comparison between model types showed that the ridge regression models with early-CRT and pre-CRT plus early-CRT showed the highest performance (ridge model with early-CRT: AUROC 0.6322 and AUPRC 0.5965, ridge model with pre-CRT plus early-CRT: AUROC 0.6151 and AUPRC 0.5757). Their sensitivity and specificity, where an optimal cut-point value was applied, are summarized in **Supplementary Table 4**. Moreover, the ridge regression models were also lower in standard deviation than other models, showing more stability to learn and predict. For the cases of using pre-CRT, all the models showed poor prediction performances. One more notable thing is that the linear model with l2 regularization showed better performance for the prediction task than the non-linear models such as tree-based ensemble or neural network. In our experiments, the best model was the ridge regression model with early-CRT. The statistics of selected features in the 1,000 best models are described in **Supplementary Table 5**. *Early-CRT PLT*, *early-CRT monocyte count*, *early-CRT PCT*, *early-CRT WBC count*, and *early-CRT neutrophil count* are the top 5 frequently selected features with more than 90%.

### 3.2 RPS System

**Figure 2:**
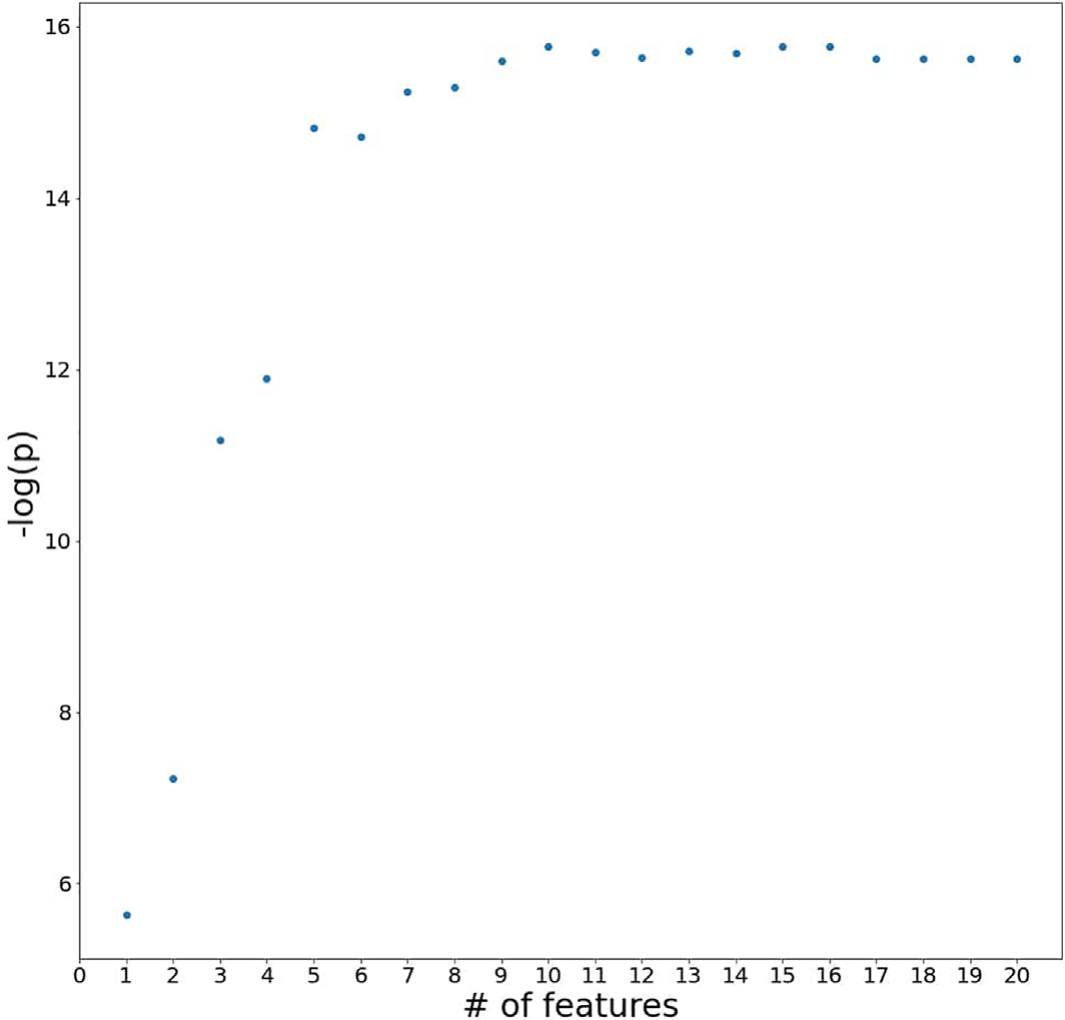
A plot of P-values for testing the difference between the scoring system and the ground truth label**’**s group by adding the blood features to the system in the most important order.

**Figure 3:**
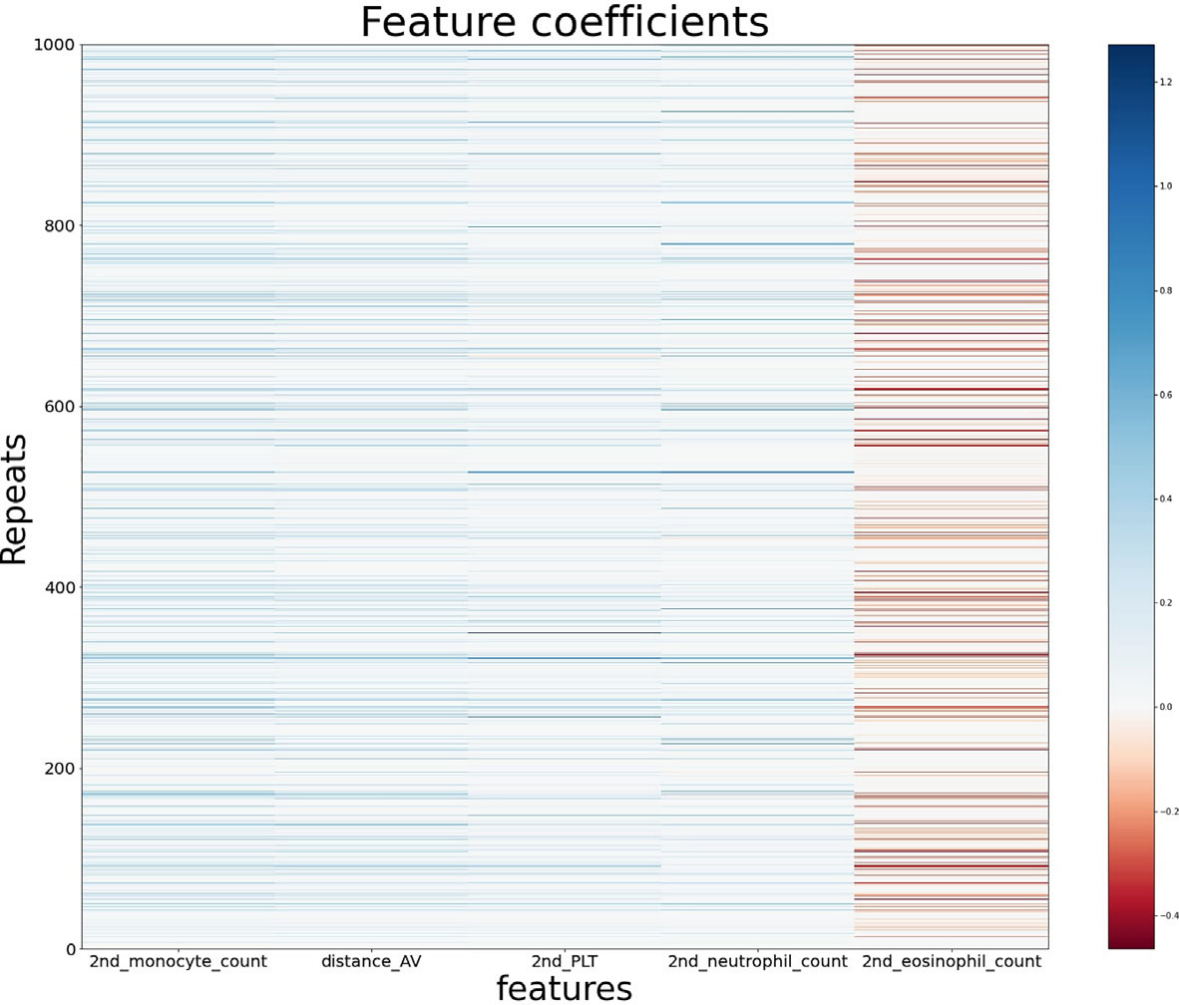
The feature coefficients of the five most important blood features in the ridge regression models using early-CRT for 1,000 repeats.

From the generalized coefficients which are calculated by an average coefficient of the 1,000 ridge regression models with early-CRT, we newly defined a scoring system for the response of preoperative CRT. The more features used in the scoring system configuration, the more significant it becomes, but the more complex it is to calculate. Namely, there exists a trade-off between simplicity and predictive power. Therefore, to find the optimal number of features for a powerful and simple scoring system, we added the features in the most important order and saw a difference in the significance between the score and the ground truth label’s group (**Figure 2**). With regards to a sign of importance, features with higher absolute values were considered as more important information. As a result, the scoring system by the combination of features with the top five highest absolute values of importance was highly significant (P-value = 3.692e−07) and a reasonably simple equation in the order of *early-CRT Monocyte count*, *Distance from anal verge*, *early-CRT PLT*, *early-CRT Neutrophil count*, and *early-CRT Eosinophil count* (Equation 5) was formed. In addition, the five features used in RPS exhibited consistent signs of coefficient throughout the 1,000 models (**Figure 3**).


(5) $$\begin{array}{c} RPS=0.000559\times early\;CRT\;Monocyte\;count+0.026172\times Distance\;from\;anal\;verge\\ +0.001021\times early\;CRT\;Platelet\;count+0.000049\times early\;CRT\;Neutrophil\;count\\ -0.000433\times early\;CRT\;Eosinophil\;count \end{array}$$

**Figure 4:**
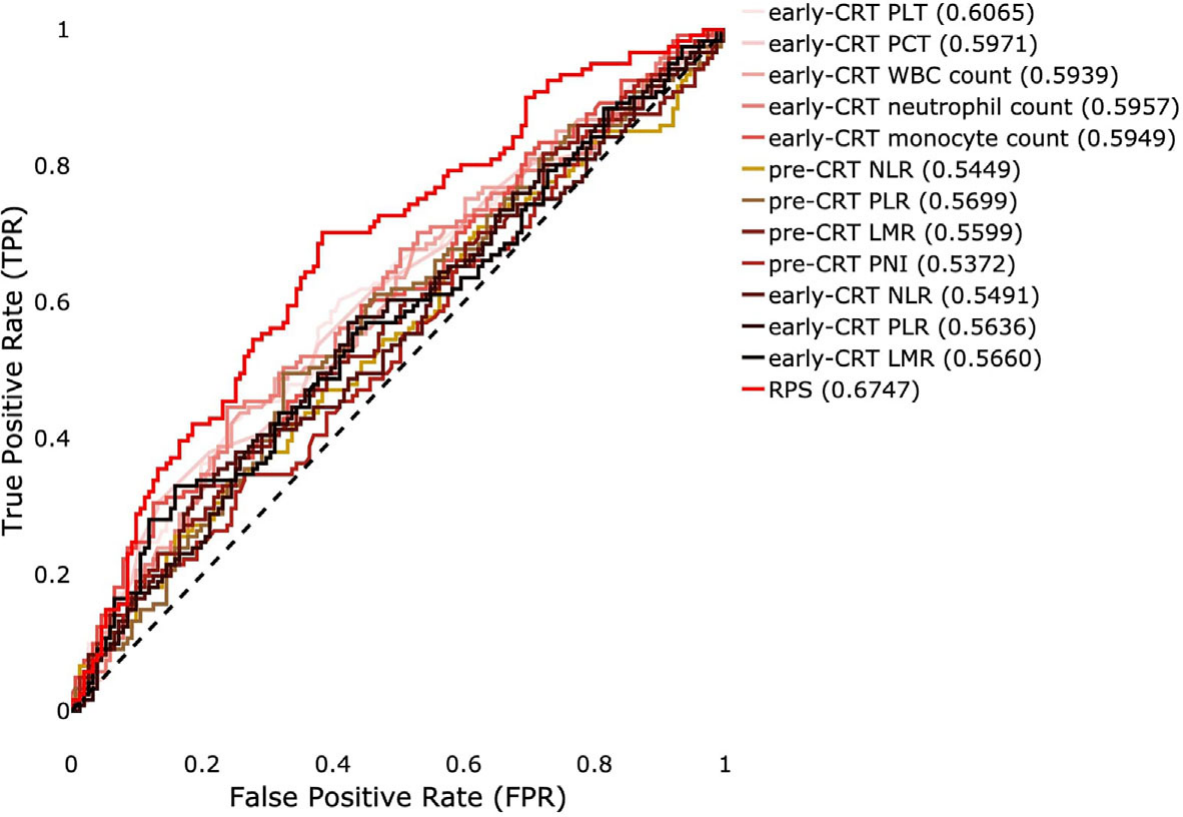
ROC curves of significant single blood features and systemic inflammatory and nutritional indicators of pre- or early-CRT and the RPS system for binary prediction for the true label. A value in parentheses means AUROC.

To verify the predictive ability of the RPS system, we compared single blood features and systemic inflammatory and nutritional indicators for whole samples. Among the features in early-CRT, early-CRT PLT, early-CRT PCT, early-CRT WBC count, early-CRT neutrophil count, early-CRT monocyte count, and early-CRT monocyte count were shown to be significant with P-value less than 0.005 (**Supplementary Tables 1** and **2**). Pre-CRT NLR, pre-CRT PLR, pre-CRT LMR, and pre-CRT PNI were obtained in pre-CRT, and available indicators early-CRT NLR, early-CRT PLR, and early-CRT LMR in early-CRT were also obtained except early-CRT PNI. **Figure 4** shows ROC curves and AUROCs of all indicators and our RPS system for the response prediction. For pre-CRT LMR and early-CRT LMR, they had the result of -pre-CRT LMR and -early-CRT LMR because of the minus correlation with the label. Among single blood features, early-CRT PLT showed the best performance with AUROC of 0.6065, and PLR showed the best performance with AUROC of 0.5699 among systematic inflammatory and nutritional markers. However, RPS showed the best predictive marker with a much higher performance AUROC of 0.6747.

**Figure 5:**
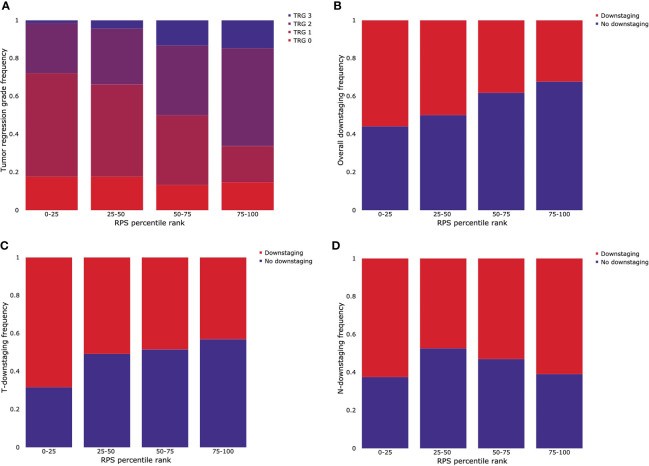
Stratification of outcomes for CRT by quartile grouping using the RPS system. **(A)** Tumor regression grade (TRG), **(B)** overall downstaging, **(C)** T-downstaging, and **(D)** N-downstaging.

The patients were grouped into four RPS groups according to the quartile. The distribution of outcomes for CRT in each group is shown in **Figure 5**. The first quartile, median, and third quartile, which are 0.5007, 0.6152, and 0.7347 respectively, were used as thresholds. For TRG, patients who were involved in a higher quantile group of RPS were likely to have lower rates of response for radiotherapy (**Figure 5A**). For overall downstaging, patients who were involved in a higher quantile group of RPS showed a lower frequency of downstaging (**Figure 5B**). The rates of T-downstaging increased in a higher quantile group of RPS. However, this trend was not shown in N-downstaging (**Figures 5C, D**).

## 4 Discussion

### 4.1 The Utility of Early-Treatment Blood Features for CRT Response Prediction

This study demonstrated that early-CRT blood features had better predictive value than pre-CRT or pre-CRT plus early-CRT in LARC. During CRT, immune cell composition of blood changes in rectal cancer (10). At the initial two weeks of CRT, white blood cell, neutrophil, lymphocyte, and monocyte counts decrease. These changes may reflect the response of CRT. Pre-treatment immune cell compositions were not associated with the response of CRT (10). However, total leukocyte, neutrophil, and monocyte counts at two weeks after initiating CRT were related to the response of CRT. In contrast, lymphocyte counts before CRT and two weeks after CRT were not different according to the response of CRT. Like these results, early-CRT immune cell compositions were associated with the response of CRT in this study (**Supplementary Table 2**). Pre-CRT immune cell compositions were not related to the response of CRT (**Supplementary Tables 1** and **2**) These results suggest that blood features during CRT can more reflect the reaction to CRT than pre-treatment blood features.

The RPS system using early-CRT blood features can be used for the prediction of preoperative CRT response in LARC. The components of the RPS system were monocyte, platelet, neutrophil, and eosinophil counts and distance from the anal verge. In other studies, these components were suggested as predictors for the response of CRT. Monocyte and neutrophil count two weeks after CRT were lower in patients with a pathologic complete response than in those without a pathologic complete response (10). Neutrophils promote tumor resistance to radiotherapy (19). Elevated platelet count before CRT was related to poor response for CRT (20–22). Platelets can affect the response of CRT by contributing to the protection of tumor cells from immune cells, stimulation of angiogenesis, and extravasation of tumor cells (23, 24). Tumors located above 5 cm from the anal verge were associated with higher rates of a complete pathological response (25). The susceptibility to CRT in high rectal cancer might be higher than that in low rectal cancer by different tumor biology.

The RPS system has the utility of an assisting role in practice. It can be used to assist the clinician’s decision to “continue or stop” at the early phase during CRT. For patients whose condition seems not to improve at 1 or 2 weeks of CRT and if their RPS is high, it may help determine “stop” for clinicians. Likewise, for patients whose condition seems to improve at 1 or 2 weeks of CRT and if their RPS is low, it may support clinicians’ decision to “continue”.

### 4.2 The Performance of Other Blood Features

Several systemic inflammatory and nutritional indicators were investigated to predict the response to CRT in rectal cancer. The results of these indicators were conflicted. First, NLR was a significant predictor of complete pathological response in multivariate analysis (8). However, NLR did not correlate with a good response in other studies. Low NLR was related to an increased likelihood of a complete pathological response only in clinical stage III (26). Second, PLR during CRT and change of PLR during CRT were significant predictors for complete pathological response (27). However, PLR did not distinguish complete regression from the residual disease in a study with 984 patients (28). In addition, NLR and PLR were not predictive of pathologic complete response in a large study including 1,527 patients (29). Third, a higher LMR was associated with good response (30). However, LMR was not an independent predictor for the response. In a previous study with 984 patients, LMR was not related with total regression (28). Lastly, pre-treatment PNI was significantly correlated with response to CRT (9, 30). In other studies, PNI did not give information of prediction for chemo- or radiotherapy (31). These discrepancies imply that these indicators may be unstable for the prediction of the response for CRT. In the present study, the performance of the RPS system was better than those of NLR, PLR, LMR, and PNI.

CEA is a broadly used tumor marker for survival prediction and post-treatment follow-up in colorectal cancer. CEA has been suggested as a predictive marker for response of chemoradiotherapy in rectal cancer. Several studies reported that elevated pre-CRT CEA level was associated with poor response to CRT (32, 33). Low post-CRT CEA level was suggested as a predictor of pathologic complete response. Although CEA can be utilized as a single measurement for prediction of response, CEA was not included as a component of the RPS system in this study.

### 4.3 Understanding the Result of the Machine Learning Approach

According to our experimental results, the linear model with l2 regularization showed better performance for the prediction task than the non-linear models such as tree-based ensemble or neural network. The AUROCs of the training set of the non-linear models, especially tree-based models, were very high. This was due to the models being highly overfitted even though the constraints were applied through the hyperparameter space to prevent overfitting. Therefore, we believe the linear model is more eligible as a predictor and we decided to construct the scoring system through the ridge model that shows the best performance with the most reasonable performance.

Most models trained on pre- plus early-CRT had the tendency to be overfitted, despite hyperparameter optimization. It causes performance differences with the models with early-CRT. This seems to be due to the presence of pre-CRT. Given that the performances were extremely low when only pre-CRT was trained alone, pre-CRT does not seem to contain important information in terms of CRT response prediction. Thus, the parameter space of pre- plus early-CRT is rather unnecessarily wider than that of early-CRT alone. Therefore, it makes the optimization problem more difficult.

### 4.4 Strengths and Limitations

Most of the previous studies have only analyzed pre-treatment blood features, but we collected both pre-treatment and early-treatment data and analyzed them together. The experiments showed that early-treatment blood features contain more important information. Furthermore, whereas the previous studies have shown significance for a single feature or the significance of existing indicators, we proposed an RPS system by applying a machine learning approach to increase the prediction performance as much as possible by considering multiple variables. The machine learning approach can prevent model overfitting through data splitting as well.

Another advantage of the RPS system is the stratification of outcomes for CRT, TRG, overall downstaging, and T-downstaging. The stratification of N-downstaging was not achieved using the RPS system. The reason of these results may be that the RPS system was derived from the response of the main tumor, not the response of lymph nodes. In patients with low RPS, the tumor is likely to downstage and respond effectively to CRT. These results suggest that the RPS system can be useful in application of daily practice.

However, our study has a few limitations. First, the number of samples used in the analysis was relatively small compared to the number of samples we collected. It is because we only used the samples that had pre- and early-treatment data, and the samples with only pre-treatment information were not used. Second, the dataset we collected contained single-center data. For modeling enhancement, increasing the number of samples and sharing of data are necessary. Further validation will be extended to multi-center data analysis. Third, the performance of our system is too low to be used in practice. Nevertheless, as shown in **Figure 5**, the obvious tendency between RPS and down staging is encouraging. We emphasize that the RPS system is easily obtained from laboratory values that are routinely measured and is superior to other previous indicators.

## 5 Conclusion

In this study, we analyzed pre-CRT and early-CRT data obtained from 272 patients and proposed the RPS system for the response of preoperative CRT in LARC. We showed that the RPS system is more predictive than the systemic inflammatory and nutritional indicators that were introduced for the same target in previous studies, and it is also a good indicator of prognostic measures. To develop a more practical predictive scoring system, it is necessary to collect more data, not just pre-treatment blood data but also early-treatment blood data in the future. Furthermore, in-depth research on the change in blood features according to CRT progress is needed to predict the response of CRT.

## Data Availability Statement

The raw data supporting the conclusions of this article will be made available by the authors, without undue reservation.

## Ethics Statement

The studies involving human participants were reviewed and approved by the Institutional Review Board, Seoul National University Hospital Biomedical Research Institute. Written informed consent for participation was not required for this study in accordance with the national legislation and the institutional requirements.

## Author Contributions

JP and DK: study design and guidance, drafting and revision of the manuscript, and study supervision. JK: data analysis, evaluation of the results, and drafting and revision of the manuscript. K-AS: study design and guidance, drafting and revision of the manuscript. MK, S-BR, S-YJ, KP, H-CK, and EC: data acquisition, analysis, and interpretation and revision of the manuscript. S-HJ: interpretation and revision of the manuscript. All authors contributed to the article and approved the submitted version.

## Funding

This study was supported by grant no. 0420180600 from the SNUH Research Fund.

## Conflict of Interest

The authors declare that the research was conducted in the absence of any commercial or financial relationships that could be construed as a potential conflict of interest.

## Publisher’s Note

All claims expressed in this article are solely those of the authors and do not necessarily represent those of their affiliated organizations, or those of the publisher, the editors and the reviewers. Any product that may be evaluated in this article, or claim that may be made by its manufacturer, is not guaranteed or endorsed by the publisher.
