# Supplementary material for: A Novel Scoring System for Response of Preoperative Chemoradiotherapy in Locally Advanced Rectal Cancer Using Early-Treatment Blood Features Derived From Machine Learning
Source: Front Oncol. 2021 Nov 29;11:790894. doi: 10.3389/fonc.2021.790894 (PMC8666428; doi:10.3389/fonc.2021.790894)
Supplement: Supplementary file 1 [file DataSheet_1.pdf]

Supplementary Material

Table S1. Clinical features and pre-CRT blood features. (ASA, American Society of Anesthesiologists score; BMI, Body mass index; Distance AV, Distance from anal verge; CEA, Carcinoembryonic Antigen; RBC, red blood cell (RBC) count; HB, Hemoglobin levels; Hct, Hematocrit (Hct) Levels; MCV, mean corpuscular volume; MCH, Mean corpuscular hemoglobin; MCHC, Mean corpuscular hemoglobin concentration; RDW, Red cell distribution width; PLT, 0.001*Platelet count; PCT, Plateletcrit; MPV, Mean platelet volume; PDW, Platelet volume distribution width; WBC count, White Blood Cell Count; neutrophil count, Neutrophil count; lymphocyte count, Lymphocyte count; monocyte count, Monocyte count; eosinophil count, Eosinophil count; basophil count, Basophil count)

Features	Tumor regression grade		P-value
	Good responder n=121	Poor responder n=151	
**Age**	59.0 ± 10.4	59.5 ± 10.5	0.296
**Sex**			0.915
Male	41 (33.9%)	59 (39.1%)	
Female	80 (66.1%)	92 (60.9%)	
**ASA**			0.902
Systemic disease	81 (66.9%)	92 (60.9%)	
Healthy	40 (33.1%)	59 (39.1%)	
**BMI**	23.5 ± 3.1	23.5 ± 3.1	0.431
**Diabetes**	20 (16.5%)	25 (16.6%)	0.999
**Hypertension**	36 (29.8%)	45 (29.8%)	0.999
**Smoking**	23 (19.0%)	21 (13.9%)	0.883
**Alcohol**	32 (26.4%)	31 (20.5%)	0.884
**Distance AV**	5.5 ± 3.0	4.8 ±2.9	0.032 (*)
**Grade**			0.779
High	14 (11.6%)	8 (5.3%)	
Low	107 (88.4%)	143 (94.7%)	
**CEA**	5.8 ± 8.5	5.6 ± 11.5	0.291
**RBC**	4.4 ± 0.5	4.5 ± 0.5	0.292
**HB**	13.3 ± 1.8	13.4 ± 1.8	0.214
**Hct**	39.9 ± 4.6	40.3 ± 4.8	0.204
**MCV**	90.7 ± 6.1	90.6 ± 6.2	0.457
**MCH**	30.0 ± 2.6	30.1 ± 2.7	0.489
**MCHC**	33.1 ± 1.3	33.2 ± 1.3	0.224
**RDW**	13.7 ± 2.7	13.3 ± 1.7	0.168
**PLT**	265.7 ± 76.0	253.1 ± 71.4	0.140
**PCT**	0.2 ± 0.1	0.2 ± 0.1	0.134
**MPV**	9.1 ± 1.4	9.1 ± 1.3	0.392
**PDW**	13.7 ± 2.9	13.5 ± 2.9	0.353
**WBC count**	7.1 ± 2.3	7.1 ± 2.0	0.446
**Neutrophil count**	4359.8 ± 1914.5	4227.7 ± 1621.2	0.249
**Lymphocyte count**	2046.2 ± 745.7	2099.6 ± 634.6	0.109
**Monocyte count**	521.2 ± 187.7	496.0 ± 156.9	0.178
**Eosinophil count**	164.2 ± 128.5	204.8 ± 178.7	0.033
**Basophil count**	42.1 ± 29.5	39.2 ± 24.5	0.297

p-value < 0.05 (*), < 0.01 (**), < 0.005 (***)

Table S2. pre-CRT and early-CRT blood features. (Calcium, serum Calcium level; Phosphorus, serum Phosphorus level; glucose, serum glucose level; BUN, serum Blood Urea Nitrogen level; uric acid, serum uric acid level; Cholesterol, serum Cholesterol level; Protein total, serum total protein level; Albumin serum Albumin, level; Bilirubin total, serum total Bilirubin level; Alkaline phosphatase, serum Alkaline phosphatase level; GOT AST, serum aspartate aminotransferase (AST) level; GPT ALT, serum alanine aminotransferase (ALT) level; creatinine, serum creatinine level;)

Features	Tumor regression grade		P-value
	Good responder n=121	Poor resPponder n=151	
**Calcium**	9.2 ± 0.5	9.2 ± 0.5	0.332
**Phosphorus**	3.5 ± 0.5	3.5 ± 0.5	0.417
**Glucose**	117.3 ± 47.5	110.6 ± 38.0	0.028 (*)
**BUN**	13.5 ± 4.5	13.7 ± 4.8	0.413
**Uric acid**	4.8 ± 1.3	5.1 ± 1.5	0.205
**Cholesterol**	179.7 ± 45.5	180.2 ± 32.8	0.233
**Protein total**	7.1 ± 0.6	7.1 ± 0.6	0.382
**Albumin**	4.2 ± 0.4	4.2 ± 0.3	0.107
**Bilirubin total**	0.7 ± 0.3	0.7 ± 0.3	0.305
**Alkaline phosphatase**	70.3 ± 20.7	72.6 ± 20.6	0.156
**GOT AST**	21.3 ± 9.6	21.4 ± 11.8	0.380
**GPT ALT**	19.6 ± 14.1	20.8 ± 16.6	0.364
**Creatinine**	0.8 ± 0.2	0.9 ± 0.3	0.096
**2nd RBC**	4.2 ± 0.5	4.2 ± 0.4	0.491
**2nd HB**	12.7 ± 1.6	12.8 ± 1.6	0.271
**2nd Hct**	37.8 ± 4.5	39.2 ± 4.4	0.378
**2nd MCV**	90.0 ± 5.6	89.9 ± 5.7	0.444
**2nd MCH**	30.1 ± 2.4	30.1 ± 2.4	0.449
**2nd MCHC**	33.4 ± 1.2	33.5 ± 1.3	0.195
**2nd RDW**	14.1 ± 3.4	13.9 ± 2.3	0.456
**2nd PLT**	209.8 ± 63.2	188.2 ± 54.6	0.001 (***)
**2nd PCT**	0.2 ± 0.1	0.2 ± 0.1	0.003 (**)
**2nd MPV**	8.9 ± 1.3	9.0 ± 1.2	0.322
**2nd PDW**	11.8 ± 3.0	11.5 ± 2.9	0.200
**2nd WBC count**	4.7 ± 1.3	4.3 ± 1.3	0.004 (**)
**2nd Neutrophil count**	2888.0 ± 1093.6	2582.2 ± 1024.9	0.003 (**)
**2nd Lymphocyte count**	1134.9 ± 454.5	1099.6 ± 399.8	0.341
**2nd Monocyte count**	439.1 ± 167.2	380.3 ± 141.2	0.004 (**)
**2nd Eosinophil count**	164.7 ± 101.6	189.0 ± 122.4	0.068
**2nd Basophil count**	21.1 ± 15.3	18.8 ± 13.3	0.083

p-value < 0.05 (*), < 0.01 (**), < 0.005 (***)

Table S3. Hyperparameter search space for six kinds of machine learning model

Model	Hyperparameter search space
Logistic regression	None
Ridge regression	C: [1e-6,1e-5,1e-4,0.0001, 0.0003, 0.001, 0.003, 0.01, 0.03, 0.1, 0.3, 1, 3, 10]
Lasso regression	C: [1e-6,1e-5,1e-4,0.0001, 0.0003, 0.001, 0.003, 0.01, 0.03, 0.1, 0.3, 1, 3, 10]
Gradient boosting	max_depth: ['auto', 'sqrt', 'log2'], max_features: [2,3,4,5,6,7,8,9,10]
Random forest	max_depth: ['auto', 'sqrt', 'log2'], max_features: [2,3,4,5,6,7,8,9,10]
2-layer neural network	hidden_layer_sizes: [2,3,4,5,6,7,8,9,10,15,20]

Table S4. Sensitivity and Specificity of total 18 models for 1,000 times repeats

	Training set		Tuning set		Validation set	
	Sensitivity (mean ± std)	Specificity (mean ± std)	Sensitivity (mean ± std)	Specificity (mean ± std)	Sensitivity (mean ± std)	Specificity (mean ± std)
**Tumor-related clinical only**						
Logistic regression	0.5850 ± 0.0194	0.5853 ± 0.0194	0.5383 ± 0.0784	0.5371 ± 0.0845	0.5314 ± 0.0725	0.5315 ± 0.0807
Ridge regression	0.5851 ± 0.0193	0.5854 ± 0.0191	0.5391 ± 0.0780	0.5375 ± 0.0839	0.5303 ± 0.0717	0.5308 ± 0.0808
Lasso regression	0.4198 ± 0.2435	0.6985 ± 0.1759	0.4310 ± 0.2545	0.6810 ± 0.1955	0.3902 ± 0.2325	0.6392 ± 0.2194
Gradient boosting	0.9130 ± 0.1078	0.8702 ± 0.0821	0.5571 ± 0.0806	0.5545 ± 0.0823	0.5016 ± 0.0775	0.4994 ± 0.0780
Random forest	0.7871 ± 0.1287	0.7876 ± 0.1292	0.5627 ± 0.0777	0.5618 ± 0.0830	0.5056 ± 0.0775	0.5039 ± 0.0793
2-layer neural network	0.5662 ± 0.0723	0.5753 ± 0.0705	0.5810 ± 0.0976	0.5893 ± 0.0952	0.5129 ± 0.0957	0.5214 ± 0.1024
**Clinical only**						
Logistic regression	0.6147 ± 0.0257	0.6147 ± 0.0265	0.5189 ± 0.0836	0.5182 ± 0.0840	0.5133 ± 0.0798	0.5125 ± 0.0829
Ridge regression	0.6137 ± 0.0268	0.6133 ± 0.0279	0.5279 ± 0.0837	0.5276 ± 0.0882	0.5116 ± 0.0781	0.5105 ± 0.0854
Lasso regression	0.4110 ± 0.2688	0.7228 ± 0.1824	0.4026 ± 0.2667	0.6963 ± 0.2067	0.3509 ± 0.2353	0.6472 ± 0.2384
Gradient boosting	0.9845 ± 0.0431	0.9844 ± 0.0434	0.5743 ± 0.0823	0.5718 ± 0.0793	0.5115 ± 0.0842	0.5097 ± 0.0858
Random forest	0.9559 ± 0.0768	0.9560 ± 0.0772	0.5724 ± 0.0824	0.5730 ± 0.0841	0.5293 ± 0.0813	0.5287 ± 0.0835
2-layer neural network	0.7483 ± 0.1378	0.7468 ± 0.1399	0.5843 ± 0.0804	0.5818 ± 0.0779	0.5036 ± 0.0862	0.5029 ± 0.0891
**Clinical + pre-CRT**						
Logistic regression w/ FS	0.6037 ± 0.0308	0.6037 ± 0.0304	0.4915 ± 0.0849	0.4895 ± 0.0874	0.4934 ± 0.0868	0.4916 ± 0.0874
Ridge regression w/ FS	0.6024 ± 0.0303	0.6028 ± 0.0305	0.4959 ± 0.0849	0.4952 ± 0.0844	0.4908 ± 0.0869	0.4900 ± 0.0878
Lasso regression	0.5448 ± 0.2755	0.7435 ± 0.1360	0.4571 ± 0.2357	0.6553 ± 0.1826	0.3992 ± 0.2111	0.5977 ± 0.2145
Gradient boosting w/ FS	0.9672 ± 0.0656	0.9667 ± 0.0677	0.5582 ± 0.0848	0.5576 ± 0.0835	0.4947 ± 0.0878	0.4949 ± 0.0861
Random forest w/ FS	0.8982 ± 0.1267	0.8980 ± 0.1261	0.5377 ± 0.0847	0.5399 ± 0.0849	0.4906 ± 0.0855	0.4907 ± 0.0859
2-layer neural network	0.6503 ± 0.1321	0.6496 ± 0.1323	0.5661 ± 0.0913	0.5658 ± 0.0900	0.4950 ± 0.1024	0.4928 ± 0.1033
**Clinical + early-CRT**						
Logistic regression w/ FS	0.6368 ± 0.0286	0.6366 ± 0.0282	0.5725 ± 0.0881	0.5751 ± 0.0840	0.5855 ± 0.0853	0.5860 ± 0.0809
Ridge regression w/ FS	0.6262 ± 0.0248	0.6261 ± 0.0257	0.6129 ± 0.0786	0.6153 ± 0.0796	**0.6094 ± 0.0806**	**0.6095 ± 0.0806**
Lasso regression	0.6161 ± 0.1523	0.6708 ± 0.0866	0.5804 ± 0.1598	0.6378 ± 0.1166	0.5468 ± 0.1538	0.6002 ± 0.1257
Gradient boosting w/ FS	0.9739 ± 0.0464	0.9742 ± 0.0462	0.5785 ± 0.0818	0.5814 ± 0.0852	0.5213 ± 0.0873	0.5204 ± 0.0905
Random forest w/ FS	0.7983 ± 0.1242	0.7986 ± 0.1243	0.5984 ± 0.0788	0.6009 ± 0.0876	0.5691 ± 0.0816	0.5674 ± 0.0917
2-layer neural network	0.6542 ± 0.0924	0.6567 ± 0.0905	0.6207 ± 0.0839	0.6265 ± 0.0843	0.5691 ± 0.0915	0.5745 ± 0.0946
**Clinical + pre- and early-CRT**						
Logistic regression w/ FS	0.6552 ± 0.0282	0.6551 ± 0.0287	0.5572 ± 0.0876	0.5569 ± 0.0819	0.5621 ± 0.0862	0.5621 ± 0.0815
Ridge regression w/ FS	0.6436 ± 0.0259	0.6433 ± 0.0250	0.6006 ± 0.0880	0.5990 ± 0.0844	0.5922 ± 0.0845	0.5944 ± 0.0829
Lasso regression	0.6151 ± 0.1905	0.6956 ± 0.1055	0.5485 ± 0.1777	0.6322 ± 0.1301	0.5105 ± 0.1729	0.5906 ± 0.1474
Gradient boosting w/ FS	0.9880 ± 0.0281	0.9879 ± 0.0286	0.5980 ± 0.0784	0.6009 ± 0.0806	0.5417 ± 0.0890	0.5441 ± 0.0913
Random forest w/ FS	0.8834 ± 0.1121	0.8831 ± 0.1126	0.6048 ± 0.0842	0.6049 ± 0.0814	0.5719 ± 0.0827	0.5700 ± 0.0867
2-layer neural network	0.7433 ± 0.1263	0.7416 ± 0.1273	0.6129 ± 0.0828	0.6143 ± 0.0811	0.5483 ± 0.0925	0.5505 ± 0.0932

Table S5. Statistics of selected features in the 1,000 ridge regression with early-CRT models

Feature	Count of selections	Feature	Count of selections
2nd PLT	983	2nd HB	88
2nd monocyte count	962	Age	75
2nd PCT	952	CEA	58
2nd WBC count	944	2nd MPV	57
2nd neutrophil count	942	2nd lymphocyte count	38
Distance AV	710	2nd Hct	30
2nd eosinophil count	505	2nd RDW	24
2nd basophil count	429	BMI	23
2nd MCHC	158	2nd MCV	23
2nd PDW	151	2nd MCH	18
		2nd RBC	14

* ”2nd” denotes early-CRT feature.
